# Teaching Safety, Saving Skin: Preventing Childhood Hot Water Burns Through Caregiver Education in Soweto, South Africa

**DOI:** 10.1002/wjs.70515

**Published:** 2026-07-27

**Authors:** Nirav Patel, Sarah Downs, Siobhan Johnstone, Sinalo Gqunu, Takwanisa Machemedze, Mbali Twala, Maatla Temane, Nomasonto Radebe, Nqaba Mthimkulu, Sello Mokoena, Ziyaad Dangor

**Affiliations:** ^1^ Department of Paediatric Surgery Chris Hani Baragwanath Academic Hospital University of the Witwatersrand Johannesburg South Africa; ^2^ Faculty of Health Sciences Medical Research Council Vaccines and Infectious Diseases Analytics Research Unit University of the Witwatersrand Johannesburg South Africa

**Keywords:** accident prevention, burns, child, global health, health education

## Abstract

**Background:**

Though preventable, burns are the leading cause of trauma‐related death in children under 4 years in South Africa and account for almost half of all pediatric trauma cases at Chris Hani Baragwanath Academic Hospital. We investigated the impact of an education‐based pediatric hot water burns prevention program on the knowledge and behavior of caregivers in Soweto.

**Methods:**

We conducted a quasi‐experimental nonequivalent controlled before‐and‐after intervention study with a 7‐month follow‐up. Households were randomly selected within purposively identified high‐burden burns clusters. At baseline, household assessments and caregiver surveys captured burn history, burn risk, first aid knowledge, and household injury risk. Subsequently, all caregivers received structured education on pediatric burns, prevention measures, and first aid. At follow‐up, the same households and individuals were reassessed to evaluate for knowledge and behavior change.

**Results:**

A total of 242 intervention and 52 control households were assessed. There were no significant differences between the control and intervention households. Most households were severely overcrowded (70.8%, 208/294). Only 7.1% (21/294) had geysers, with 89.8% (264/294) dependent on manually boiled water for bathing, despite most households being electrified (97.9%, 288/294). Relative to baseline, there was a 30.8% (95% CI 22.2%–40.9%) increase in knowledge on the correct first aid for hot water burns postintervention. A 43.3% (95% CI 36.5%–50.5%) increase in stove tops being kept > 1.4 m off the ground, 40.3% (95% CI 30.0%–51.4%) increase in appropriately checking the temperature of bath water, 38.3% (95% CI 29.1%–48.4%) increase in ensuring pot handles are out of reach, and 23.5% (95% CI 15.8%–33.6%) decrease in handling of hot beverages while holding a child were also noted postintervention (all exact McNemar *p* < 0.001). There was no change in the number of households with childfree safe zones postintervention.

**Conclusions:**

Caregiver education was associated with significant improvements in caregiver knowledge and selected safety practices. Structural constraints, such as overcrowding, may limit the adoption of some positive behavioral changes, underscoring the need for additional system level intervention.

## Graphical abstract

Though preventable, burns are the leading cause of trauma‐related death in children under 4 years in South Africa and account for almost half of all pediatric trauma cases at Chris Hani Baragwanath Academic Hospital. We investigated the impact of an education‐based pediatric hot water burns prevention program on the knowledge and behavior of caregivers in Soweto. Caregiver education was associated with significant improvements in caregiver knowledge and selected safety practices. Structural constraints, such as overcrowding, may limit the adoption of some positive behavioral changes, underscoring the need for additional system level intervention.

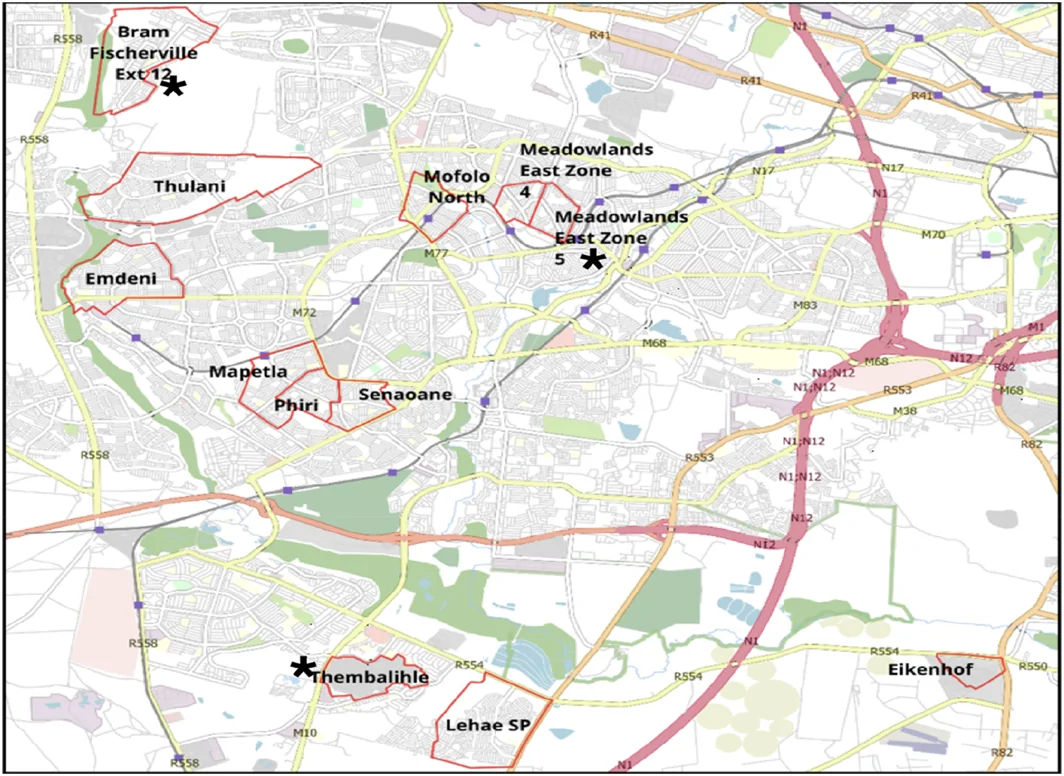

## 1 Introduction

Pediatric burns represent a significant global public health challenge. Worldwide, burns account for 10% of all childhood deaths, with the highest burden of injury in low‐ and middle‐income countries (LMICs) in Africa [1]. Pediatric burns are common, impose a substantial physical, emotional, and financial burden on victims and their families, require significant healthcare resources to manage, and are largely preventable [1, 2, 3, 4, 5]. In LMICs, the risk of pediatric burn injury is amplified by rapid urbanization, poverty, overcrowding, lack of adequate infrastructure, use of unsafe energy sources, poor parental education, and lack of appropriate law enforcement [6, 7].

In South Africa, burns are the most common cause of trauma‐related mortality in children under 4 years of age, accounting for 1300 deaths annually [2, 3]. Chris Hani Baragwanath Academic Hospital (CHBAH), a large tertiary hospital in Soweto, Johannesburg, is one of only 2 specialist pediatric burns centers in South Africa. At CHBAH, pediatric burns admissions increased by 45% from 2009 to 2019 [8, 9]. In 2019, burns were responsible for 19% of all surgical procedures and 48% of all pediatric trauma consultations, with 891 children treated for burns at CHBAH alone [9]. In South Africa, the high burden of pediatric burns is aggravated by a lack of skills and equipment to appropriately manage burns outside of large regional and tertiary centers [2].

Data reveal that most children with burns admitted to CHBAH are burnt with hot water, are males under the age of 3 years, sustain severe injury as per American Burn Society guidelines, and that approximately 40% receive either no or the incorrect first aid [10, 11]. Soweto is a large predominantly low‐income urban township characterized by high levels of overcrowding, unemployment and multiple informal settlements with poor basic infrastructure [12].

Despite the local scale and preventable nature of pediatric HWB, there are no structured pediatric burns prevention programs in Soweto. To address this gap, an education based pediatric HWB prevention program was developed and implemented in conjunction with a local nonprofit organization (Surgeons For Little Lives, SFLL) and the Vaccines and Infectious Diseases Analytics unit (VIDA) at the University of the Witwatersrand (Wits).

Given the high number of children under the age of 5 admitted with HWB and high proportion receiving incorrect or no first aid, the program aims to educate the caregivers of young children on the risk of HWB, how to mitigate this risk, and the correct first aid for HWB with a view to enhance knowledge of HWB and promote protective behavior change in the caregivers of young children. Parental knowledge is a recognized modifiable risk factor in pediatric HWB and the intervention was developed pragmatically on the basis of local data [13, 14]. The intervention consisted of repetitive structured education delivered through simple locally adapted messaging in local languages, using a combination of printed and digital media. This study aims to evaluate the impact of the educational intervention on the knowledge and behavior of caregivers of children in Soweto and Thembelihle, South Africa.

## 2 Methods

**FIGURE 1:**
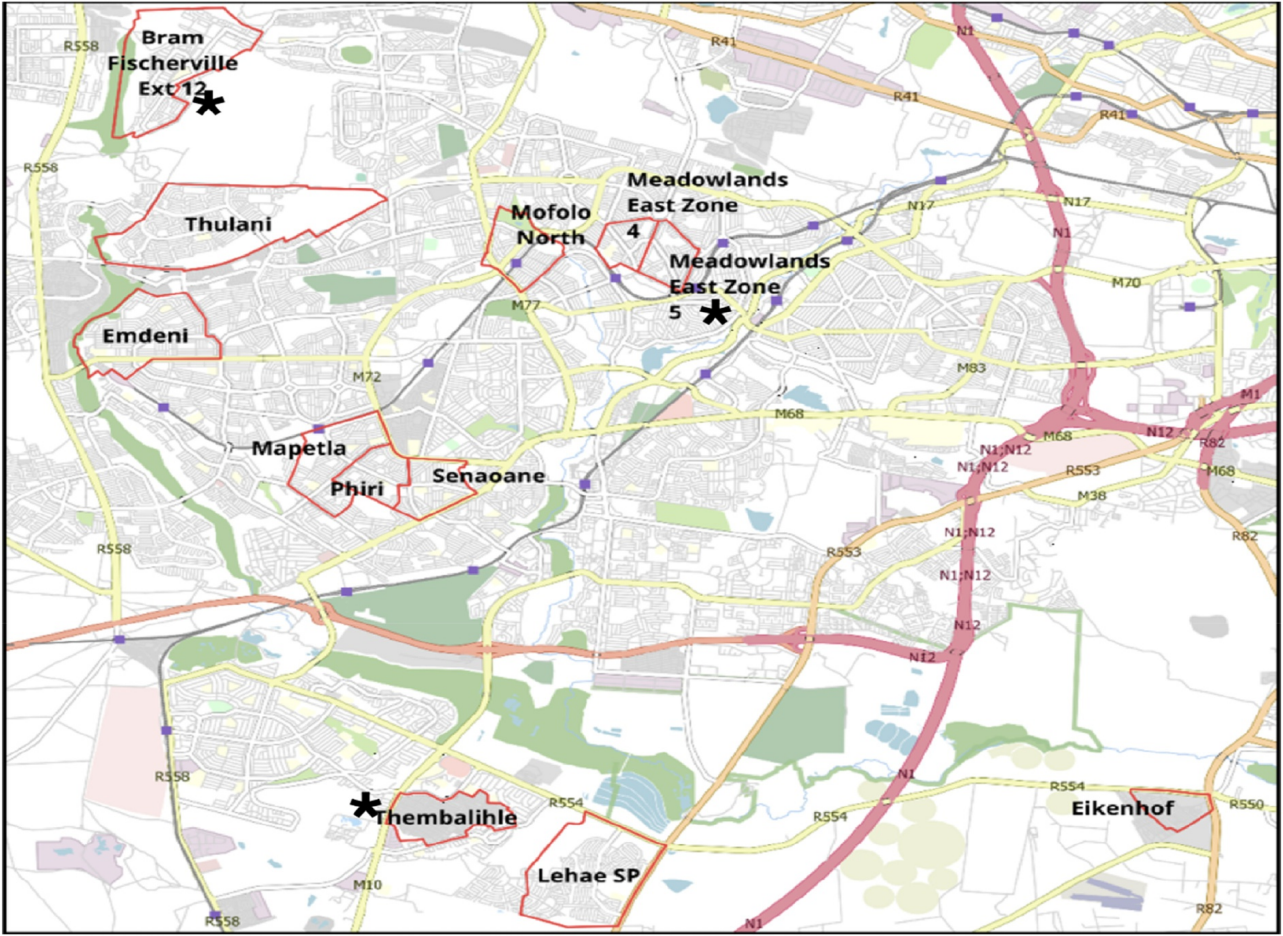
Geographic distribution of SaT‐HDSS clusters and selected study sites. Red demarcated areas represent SaT‐HDSS clusters; clusters selected for the burns intervention study are indicated by an asterisk (*).

We conducted a community‐based quasi‐experimental controlled before‐and‐after (CBA) study with a 7 month follow‐up and a nonequivalent control group receiving a delayed intervention. The study was undertaken across two suburbs (Meadowlands and Braamfischerville) of Soweto, and the neighboring settlement of Thembelihle, Gauteng, South Africa between February and September 2025 (Figure 1). All three suburbs are known high pediatric HWB prevalence areas [10].

Eligible households were identified through VIDA's existing Soweto and Thembelihle Health and Demographic Surveillance System (SaT‐HDSS). Households with at least one child under the age of 5 years and at least one caregiver of a child under 5 years present at the baseline visit were eligible for inclusion. A sample size of 388 households was required to detect a 10% change in caregiver knowledge with 80% power. To account for a 25% nonresponse rate, a total of 485 households were randomly selected.

Data were collected by SaT‐HDSS fieldworkers trained in study data collection methods and in the burns prevention intervention. Intervention households were visited twice. At the baseline visit, caregivers completed a questionnaire to assess knowledge of burns, burns risks, and first aid for HWB. Simultaneously, fieldworkers conducted an independent assessment of high‐risk household practices. These practices included the absence of a kitchen separate from the rest of the home, stove tops within 1.4 m of the ground, and electrical cables for cooking appliances within 1.4 m of the ground. Severe and extreme household overcrowding were defined using World Health Organization housing indicators: severe overcrowding as > 2 persons per sleeping room, and extreme overcrowding as > 3 persons per sleeping room [15]. Child safe zones were defined as designated areas of the home that children were never allowed to enter.

Following baseline data collection and assessment, caregivers received feedback on high‐risk household practices and structured education. Structured education included sensitization of caregivers to the risk childhood HWB, specific HWB prevention measures that can be adopted in the home, and education on appropriate and inappropriate HWB first aid. Importantly, this process involved direct interaction between the field workers and caregivers in real time with each component of the educational intervention reviewed in person rather than fieldworkers simply leaving the educational material developed for caregivers to read themselves. Examples of the printed education materials utilized are provided as supplementary material (Supporting Information S1: Figure 1).

**FIGURE 2:**
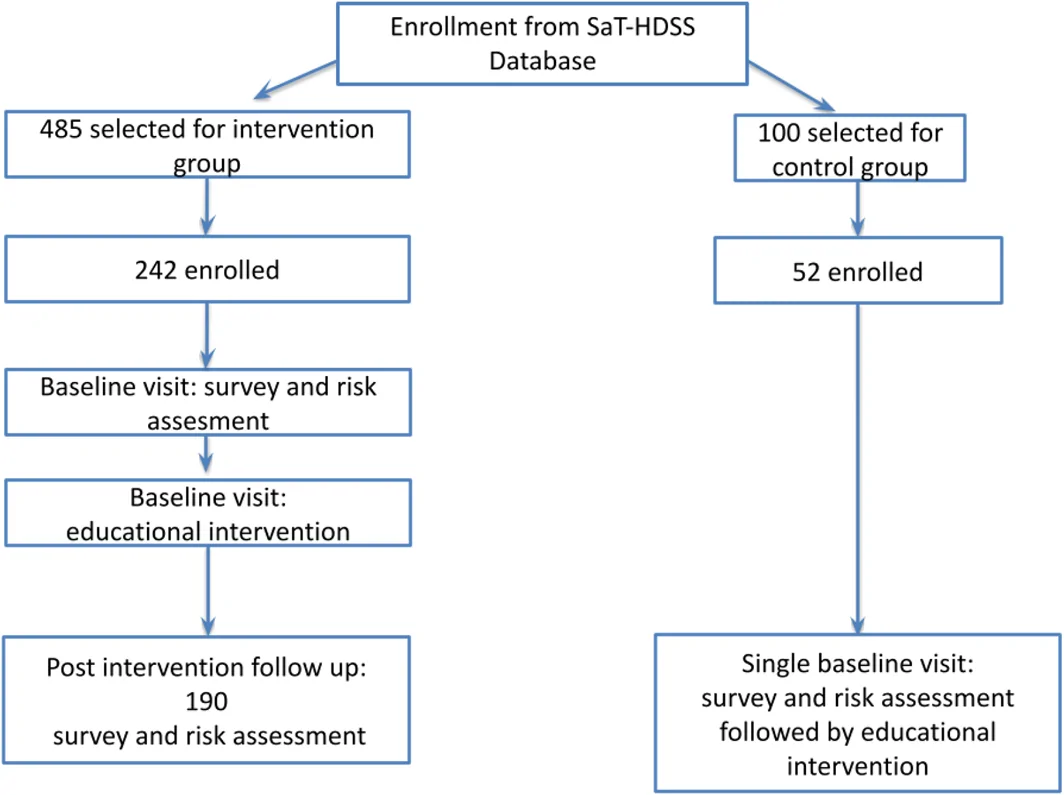
Participant flow diagram of household enrollment, allocation, and follow‐up.

A follow‐up of the same intervention households and same individual caregivers occurred 7 months after the initial visit, during which time the same knowledge‐based questionnaire and assessment of high‐risk practices survey was completed. Control households were visited once only, contemporaneously with the follow‐up visit for the intervention households. Control households were included to ensure that any observed changes in the knowledge and behavior of individuals in the intervention households were due to the intervention itself. A participant flow diagram to describe the study methodology is provided in Figure 2.

This study was reported in accordance with the Transparent Reporting of Evaluations with Nonrandomized Designs (TREND) statement [16].

### 2.1 Statistical Analysis

Categorical variables were analyzed using the chi‐squared or Fisher's exact test as appropriate. All continuous variables were reported with medians and interquartile ranges, and comparisons were made using the Mann–Whitney *U* test. Changes in burns‐related knowledge and behaviors pre and postintervention were compared at the household level. For the primary analysis, a single caregiver per household was randomly selected at each visit to ensure independence of observations and comparability across time points. Baseline and postintervention responses were matched at the household level. A sensitivity analysis was conducted restricting the sample to caregivers who were present and interviewed at both baseline and postintervention visits. In this analysis, responses were matched at the individual caregiver level to assess whether household‐level estimates may have been affected by intrahousehold variability in knowledge or behaviors between different caregivers or by differential respondent characteristics at baseline and follow‐up. Paired binary outcomes (including multiple‐response items recorded as correct/incorrect) were compared using McNemar's test. Absolute percentage point changes from baseline were reported. A *p*‐value < 0.05 was considered significant. 95% confidence intervals for the proportion of households that transitioned to safe practice or correct knowledge were calculated using the Wilson score interval. Data were analyzed using Stata version 18 (StataCorp, College Station, TX, USA).

Written informed consent was obtained from all participants. Ethical approval was obtained from the Wits Human Ethics Research Committee (240810).

## 3 Results

Over the study period, 242 households in the intervention group and 52 households in the control group were enrolled. Recruitment was constrained by limited household availability, including locked gates and the absence of a primary caregiver at the time of data collection, despite repeat visits and visits conducted outside of standard working hours for both the intervention and control groups. Nevertheless, the sample of households was sufficient to detect changes in key knowledge and behavioral outcomes. There were no missing data.

### 3.1 Demographics and Household Level Characteristics

Most households were electrified (97.9%, 288/294), overcrowded (70.7%, 208/294), classified as informal housing (72.1%, 212/294), relied on manually boiled water for bathing (89.7%, 264/294), and had similar adult/household, child/household, adult sex distribution/household, and child sex distribution/household ratios (Table 1). There were no significant demographic, knowledge, or behavioral differences noted between the intervention and control households (Supporting Information S1: Tables S1 and S2).

**TABLE 1:** Demographic and household level characteristics.

	Intervention	Control	Total	Significance
Households (*n*)	242	52	294	*p*
Formal electricity, % (*n*)	97.5 (236)	100 (52)	97.9 (288)	0.595
Severe crowding, % (*n*)	72.7 (176)	61.5 (32)	70.7 (208)	0.108
Extreme crowding, % (*n*)	33.1 (80)	21.2 (11)	30.9 (91)	0.092
Formal housing, % (*n*)	23.6 (57)	28.9 (15)	24.5 (72)	0.396
Informal housing, % (*n*)	72.3 (175)	71.2 (37)	72.1 (212)	
Adults per household (> 18 years old), median (IQR), *n*	3 (2–5), 893	3 (2–4), 186	3 (2–5), 1079	0.477
Adult sex, female, % (*n*)	84.0 (203)	90.0 (45)	84.3 (248)	0.275
Children per household (< 5 years old), median (IQR), *n*	1 (1–2), 336	1 (1–2), 76	1 (1–2), 412	0.947
Child sex, female, % (*n*)	47.3 (159)	57.5 (42)	68.4 (201)	0.114
Boil water for bathing, % (*n*)	92.3 (229)	87.5 (35)	89.7 (264)	0.304
Previous history of burns, % (*n*)	31.8 (77)	21.5 (11)	29.9 (88)	0.128

### 3.2 Risk Assessment

**FIGURE 3:**
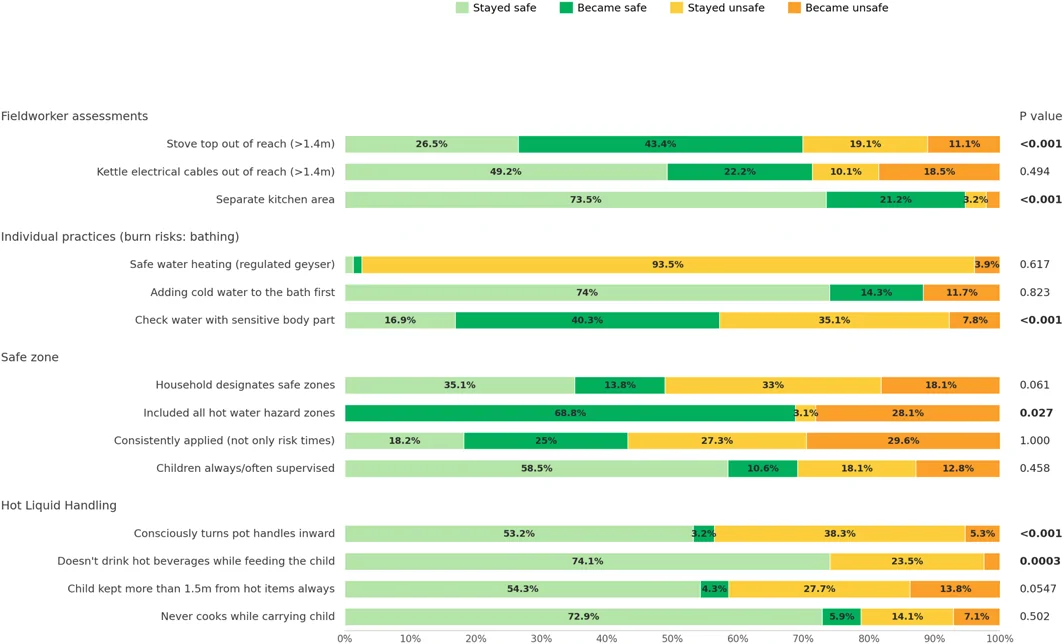
Stacked bar chart showing change in behavior from baseline at the household level. Significant changes as per McNemar's outcome in bold.

Compared with baseline, postintervention assessment showed a 43.3% (95% CI 36.5%–50.5%, *p* < 0.001) increase in stove tops being kept > 1.4 m off the ground, 40.3% (95% CI 30.0%–51.4%, *p* < 0.001) increase in appropriate checking of the temperature of bath water, a 38.3% (95% CI 29.1%–48.4%, *p* < 0.001) increase in ensuring pot handles are turned inward (out of the reach of children), and a 23.5% (95% CI 15.8%–33.6%, *p* < 0.001) reduction in caregivers handling hot beverages while feeding children (Figure 3). There were no differences in the number of households with dedicated child safe zones postintervention.

### 3.3 Knowledge Assessment

**FIGURE 4:**
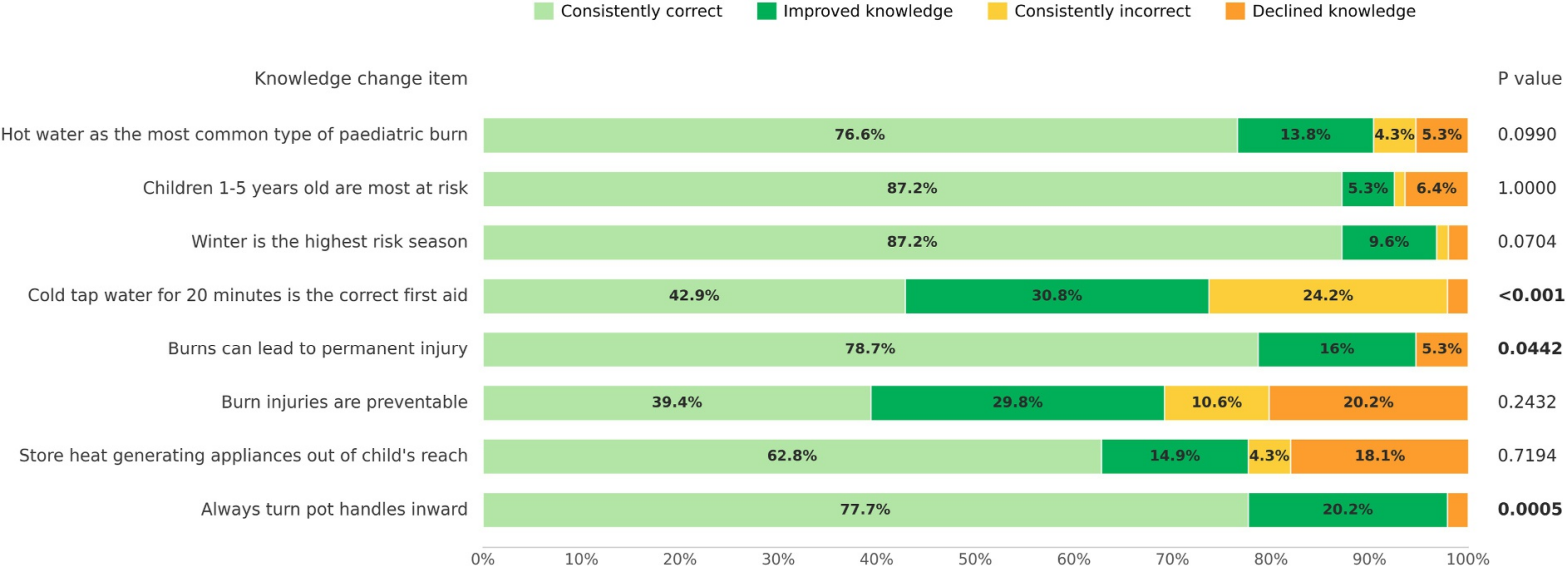
Stacked bar chart showing change in knowledge from baseline at the household level. Significant changes as per McNemar's outcome in bold.

Compared with baseline, there was a 30.8% (95% CI 22.2%–40.9% and *p* < 0.001) improvement in identification of the correct first aid for HWB (Figure 4).

Findings from the caregiver‐matched sensitivity analysis were consistent with the primary household‐level analysis, with no changes in statistical significance observed for most outcomes under behavior and knowledge assessments (Supporting Information S1: Tables S3 and S4). Data for the household level analysis are provided in Supporting Information S1: Tables S5 and S6.

### 3.4 Burn History

A total of 88 households (29.9%, 88/294) reported prior history of burns involving 113 individuals. Of the 113 individuals with prior burn history, 47.8% (54/113) were children under 5 years of age. A total of seven households (3.7%, 7/190) in the intervention group reported a new burn between visit 1 and visit 2. First aid practices in households with a new burn posteducational intervention did not differ significantly from baseline (*p* = 0.096).

## 4 Discussion

Pediatric HWB are common, devastating, and largely preventable [1, 7]. In high income countries, preventative interventions are well established and contribute to decreased burdens of burn injury relative to LMICs [17, 18]. In LMICs, the high burden of pediatric HWB and poorly functioning healthcare systems unable to manage the existing disease burden necessitate preventative interventions. Such interventions represent both an upstream attempt to decrease the overall disease burden and an attempt to improve quality of care for those children that do sustain burn injury through their impact on resource allocation. Parental knowledge is a recognized modifiable risk factor for childhood HWB that can be addressed through education campaigns [13, 14]. In this study, and consistent with international literature, we showed that educational intervention resulted in a significant improvement in knowledge with 16%–31% absolute increases in caregivers' knowledge of HWB and correct HWB first aid, and positive behavior change with 23%–43% increases in the adoption of safe behaviors [13, 14, 18]. We noted the same broad findings when knowledge and behavior change were analyzed at the household and at the caregiver matched levels. This suggests that the educational intervention was disseminated amongst caregivers in the absence of the fieldworkers. Though the mechanism of this information sharing and behavior change is not known, this finding does point to the scalability of low cost educational interventions and is indicative of a level 3 impact (learning resulting in behavior change) in the Kirkpatrick Model [19]. Given the absence of significant differences in the behavioral practices and knowledge of the intervention and control groups, we are confident that the changes in knowledge and behavior noted were due to the educational intervention alone. The effect of knowledge and behavior change on first aid practices for HWB, and the prevalence and severity of pediatric HWB (Kirkpatrick Level 4) in the study setting, is yet to be determined [19].

When burns do occur, appropriate first aid is critical to minimize adverse injury outcomes and sequelae [20]. This is particularly relevant in the local environment where appropriate burn first aid knowledge is poor, and existing practices (of applying toothpaste, butter, eggs, maize meal, and cleaning liquid to HWB) often exacerbate the injury from HWB [11]. In resource constrained LMICs, interventions to reduce demand for services may be as (if not more) impactful than interventions to increase capacity. We observed 7 new burns in the postintervention period without any statistically significant change in first aid practice. Failure to observe statistically significant changes in first aid practice may be due to the short follow‐up period and relatively low incidence of burns postintervention. Follow‐up study to assess the long term impact of the educational intervention on local first aid practices is required.

The high number of intervention and control households reporting a history of pediatric burns in the study group suggest the need for a structured systematic mechanism to report, quantify, and classify all pediatric burns in the local environment. At present, detailed data on pediatric burns are not routinely captured or shared at primary, secondary, or tertiary levels. As such, it is likely that the problem of pediatric burns in the community is greater than evidenced by the hospital level data at CHBAH. This tendency for hospital level data to underestimate the prevalence of common conditions in LMICs is well documented [21]. Establishment of a pediatric burns registry could improve surveillance, inform prevention efforts, and support health system planning [17, 22, 23, 24]. Given that interventions should be based on local data for maximum efficacy, the need for such a registry is further justified [18].

Although mostly electrified, the majority of households relied on manually boiled water (either on a stove top or kettle) for bathing. Taken together, the high level of overcrowding, the high number of informal households in the study population, the reliance on manually boiled water, and the fact that there was no significant increase in the use of household child safe zones in the postintervention period suggest that education of care givers alone may not be sufficient to comprehensively decrease pediatric HWB burn risk in the local environment. The superior efficacy of a mixed methods approach reliant on both active (which require sustained behavior change) and passive (which do not rely on host behavior) interventions in burns prevention is a feature of much of the existing literature and is likely due to the plurality of social, economic, behavioral, and other risk factors for pediatric burns [13, 17, 18, 25, 26]. In this regard, VIDA and SFLL in partnership with the Oxford University Global Surgery Group have embarked on a program to design, implement, and evaluate microinterventions to overcome structural barriers and complement the educational intervention reported to make informal settlements safer for children. The project is entitled “The Safe Mkhukhu: Preventing Childhood Injury in Informal Settlements in South Africa” and draws on the Zulu word (mkhukhu) for shack or makeshift house.

## 5 Limitations

The study had several limitations including the relatively short follow‐up period, the fact that it was restricted to three suburbs with high pediatric HWB prevalence, the small size of the control group relative to the intervention group, the fact that the final realized sample was lower than required by power calculations, and the fact that self‐reported practices may be subject to social desirability bias. Importantly, social desirability bias was somewhat mitigated by the direct assessment of key behavioral measures by trained fieldworkers.

## 6 Conclusion

Education of caregivers improved their knowledge of pediatric burns, knowledge of appropriate burn first aid, and resulted in significant positive behavior change. Structured education is a low cost, sustainable, and scalable intervention that may be replicated in similar LMIC environments. Systemic issues, such as overcrowding, may prevent the adoption of certain injury prevention behaviors. Further study is required to assess the long‐term impact of the educational intervention on pediatric HWB prevalence and severity and first aid practices in Soweto.

## Author Contributions

**Nirav Patel:** conceptualization, methodology, investigation, formal analysis, writing – original draft, writing – review and editing. **Sarah Downs:** conceptualization, methodology, investigation, data curation, supervision, software, formal analysis, writing – original draft, writing – review and editing. **Siobhan Johnstone:** conceptualization, methodology, investigation, formal analysis, writing – original draft, writing – review and editing. **Sinalo Gqunu:** conceptualization, methodology, investigation, and writing – review and editing. **Takwanisa Machemedze:** conceptualization, methodology, investigation, formal analysis, writing – review and editing. **Mbali Twala:** conceptualization, investigation, and writing – review and editing. **Maatla Temane:** conceptualization, methodology, data curation, supervision, resources, project administration, writing – review and editing, investigation. **Nomasonto Radebe:** conceptualization, methodology, investigation, and writing – review and editing. **Nqaba Mthimkulu:** conceptualization, methodology, investigation, and writing – review and editing. **Sello Mokoena:** conceptualization, methodology, investigation, and writing – review and editing. **Ziyaad Dangor:** conceptualization, methodology, investigation, supervision, formal analysis, writing – original draft, writing – review and editing.

## Funding

The authors have nothing to report.

## Conflicts of Interest

Nirav Patel is on the editorial board for the *World Journal of Surgery*. Sarah Downs, Siobhan Johnstone, Sinalo Gqunu, Takwania Machemedze, Mbali Twala, Maatla Temane, Nomasonto Radebe, Nqaba Mthimkulu, Sello Mokoena, and Ziyaad Dangor have declared no conflicts of interest. Nirav Patel is a member of the Global Initiative for Children's Surgery.

## Supporting information


Supporting Information S1

## Data Availability

The data that supports the findings of this study are available in the supplementary material of this article.
