# Supplementary material for: Teaching Safety, Saving Skin: Preventing Childhood Hot Water Burns Through Caregiver Education in Soweto, South Africa
Source: World J Surg. 2026 Jul 27;50(9):2692–8. doi: 10.1002/wjs.70515 (PMC13576568; doi:10.1002/wjs.70515)
Supplement: Supplementary file 1 [file WJS-50-2692-s001.docx]

Supplementary Figure 1. English and Zulu printed educational materials

**Supplementary Table S1. Household characteristics, demographics and burn history**

|  | **Baseline (B)** | **Control (C)** | **Post-intervention (P)** | **p-value** | | |
| --- | --- | --- | --- | --- | --- | --- |
|  |  |  |  | **B-C** | **B-P** | **C-P** |
| **Household Characteristics** | **N=242** | **N=52** | **N=190** |  |  |  |
| **Cooking or Boiling Practices:** | | | | | | |
| Formal Electricity, % (n) | 97.52 (236) | 100 (52) | 97.37 (185) | 0.595 | 1.000 | 0.588 |
| Formal Electricity Only, % (n) | 82.23 (199) | 80.77 (42) | 80.00 (152) | 0.803 | 0.555 | 0.902 |
| Unauthorized/unregulated Electricity, % (n) | 0.83 (2) | 0 | 0.53 (1) | 1.000 | 1.000 | 1.000 |
| Gas in combination, % (n) | 14.88 (36) | 17.31 (9) | 17.37 (33) | 0.659 | 0.483 | 0.992 |
| Gas only, % (n) | 1.24 (3) | 0 | 1.58 (3) | 1.000 | 1.000 | 1.000 |
| Paraffin in combination, % (n) | 2.07 (5) | 0.00 (0) | 2.11 (4) | 0.590 | 1.000 | 0.580 |
| Wood in combination, % (n) | 1.24 (3) | 1.92 (1) | 1.58 (3) | 0.543 | 1.000 | 1.000 |
| Separate kitchen area*, % (n) | 73.1 (177) | 78.9 (41) | 76.2 (144) | 0.394 | 0.471 | 0.688 |
| Stove top out of reach (>1.4m)*, % (n) | 38.0 (92) | 63.5 (33) | 69.8 (132) | **0.001** | **<0.001** | 0.381 |
| Kettle cable out of reach (>1.4m)*, % (n) | 67.36 (163) | 78.85 (41) | 71.43 (135) | 0.103 | 0.364 | 0.286 |
| **Crowding index (WHO definition, household members/sleeping room):** | | | | | | |
| Severe Crowding (Crowding >2), % (n) | 72.73 (176) | 61.54 (32) | 73.16 (139) | 0.108 | 0.920 | 0.103 |
| Extreme Crowding (Crowding >3), % (n) | 33.06 (80) | 21.15 (11) | 35.26 (67) | 0.092 | 0.631 | 0.054 |
| **Supervision index (Adults/child under 5):** | | | | | | |
| Low (<2 adult per child), % (n) | 16.12 (39) | 17.31 (9) | 15.26 (29) | 0.912 | 0.963 | 0.900 |
| Moderate (2-3 adults per child), % (n) | 52.07 (126) | 53.85 (28) | 53.16 (101) |  |  |  |
| High (>3 adults per child), % (n) | 31.82 (77) | 28.85 (15) | 31.58 (60) |  |  |  |
| **Housing Type*:** | | | | | | |
| Informal dwelling, % (n) | 23.97 (58) | 28.85 (15) | 22.11 (42) | 0.396 | 0.899 | 0.305 |
| Formal dwelling, % (n) | 72.31 (175) | 71.15 (37) | 74.21 (141) |  |  |  |
| Mixed formal & informal dwelling, % (n) | 3.72 (9) | 0 | 3.68 (7) |  |  |  |
| **Demographics — Adults enrolled** | **N=294** | **N=50** | **N=198** |  |  |  |
| Adults (>18 years old) per household, median (IQR) | 3 (2-5) | 3 (2-4) | 3 (2–5) | 0.477 | 0.396 | 0.238 |
| Adult sex, female, % (n) | 84.01 (247) | 90.00 (45) | 87.88 (174) | 0.275 | 0.232 | 0.677 |
| Adult age, years, median (IQR) | 36 (28-55) | 35 (28-42) | 39 (29-56) | 0.632 | 0.232 | 0.245 |
| **Level of education:** | | | | | | |
| No formal education, % (n) | 1.71 (5) | 2.04 (1) | - | 0.389 | - | - |
| Some primary school, % (n) | 5.82 (17) | 0 | - |  |  |  |
| Primary school (up to Grade 7), % (n) | 5.48 (16) | 4.08 (2) | - |  |  |  |
| Incomplete secondary, % (n) | 35.62 (104) | 26.53 (13) | - |  |  |  |
| Completed Grade 12/Std 10, % (n) | 44.52 (130) | 59.18 (29) | - |  |  |  |
| College/University, % (n) | 6.51 (19) | 8.16 (4) | - |  |  |  |
| Not sure, % (n) | 0.34 (1) | 0 | - |  |  |  |
| **Children (<5 years old) per household, median (IQR), n** | **1 (1–2), 336** | **1 (1–2), 73** | **1 (1–2)** | **0.947** | **0.663** | **0.739** |
| Child Age (1-5 years old), years, median (IQR), n | 3 (2-4), 300 | 3 (3-4), 66 | - | 0.054 | - | - |
| Child Age (<12 months old), months, median (IQR), n | 4 (1-7), 36 | 4 (2-7), 7 | - | 0.842 | - | - |
| Child sex, female, % (n) | 47.32 (159) | 57.53 (42) | - | 0.114 | - | - |
| **Households with Burn History#:** | **N=99** | **N=14** | **N=9** |  |  |  |
| **Burn requiring medical care (ever or since B), % (n)** | **31.82 (77)** | **21.15 (11)** | **3.68 (7)** | **0.128** | **-** | **-** |
| One burn event, % (n) | 75.32 (58) | 81.82 (9) | 85.71 (6) | 0.437 | - | - |
| Two burn events, % (n) | 20.78 (16) | 9.09 (1) | 14.29 (1) |  | - | - |
| Three burn events, % (n) | 3.9 (3) | 9.09 (1) | 0 |  | - | - |
| **Age at most recent burn:** | | | | | | |
| 0-5 years old, % (n) | 47.47 (47) | 50.00 (7) | 11.11 (1) | 0.859 | - | - |
| 6-12 years, % (n) | 15.15 (15) | 7.14 (1) | 22.22 (2) | 0.421 | - | - |
| 13-18 years, % (n) | 11.11 (11) | 21.43 (3) | 11.11 (1) | 0.273 | - | - |
| >19 years, % (n) | 26.26 (26) | 21.43 (3) | 55.56 (5) | 0.698 | - | - |
| **Burn Cause:** | | | | | | |
| Flame, % (n) | 14.14 (14) | 0 | 33.33 (3) | 0.133 | - | - |
| Flammable chemical, % (n) | 1.01 (1) | 0 | 0 | 0.706 | - | - |
| Hot water, % (n) | 63.64 (63) | 64.29 (9) | 44.44 (4) | 0.962 | - | - |
| Hot surface, % (n) | 14.14 (14) | 35.71 (5) | 22.22 (2) | **0.043** | - | - |
| Other (Porridge, Hot Oil), % (n) | 7.07 (7) | 0 | 0 | 0.304 | - | - |
| **Place where burn occurred:** | | | | | | |
| Inside home, % (n) | 91.92 (91) | 100.00 (14) | 100 (9) | 0.270 | - | - |
| Elsewhere, % (n) | 8.08 (8) | 0 (0) | 0 | - | - | - |
| **Burn First Aid given:** | | | | | | |
| No home first aid, % (n) | 27.27 (27) | 21.43 (3) | 33.33 (3) | 0.643 | 0.698 | 0.526 |
| Toothpaste, % (n) | 7.07 (7) | 7.14 (1) | 11.11 (1) | 0.992 | 0.658 | 0.742 |
| Cold /frozen item, % (n) | 3.03 (3) | 0 (0) | 0 | 0.509 | 0.596 | - |
| Oil-based substance, % (n) | 11.11 (11) | 14.29 (2) | 11.11 (1) | 0.728 | 1.000 | 0.825 |
| Food items, % (n) | 5.05 (5) | 0 (0) | 0 | 0.390 | 0.490 | - |
| Ran tap water with ICE, % (n) | 15.15 (15) | 7.14 (1) | 11.11 (1) | 0.421 | 0.744 | 0.742 |
| Tap water, % (n) | 28.28 (28) | 35.71 (5) | 55.56 (5) | 0.567 | 0.096 | 0.349 |
| Over-the-counter burn cream, % (n) | 9.09 (9) | 14.29 (2) | 0 | 0.539 | 0.345 | 0.235 |
| Not sure, % (n) | 6.06 (6) | 7.14 (1) | 0 | 0.875 | 0.447 | 0.412 |
| Other (Antiseptic, urine, aloe plant), % (n) | 7.07 (7) | 0 (0) | 0 | 0.304 | 0.409 | - |
| **Healthcare in burn case:** | | | | | | |
| Primary Health Facility / GP / Nurse, % (n) | 50.51 (50) | 42.86 (6) | 33.33 (3) | - | - | - |
| Emergency department/Casualty, % (n) | 17.17 (17) | 14.29 (2) | 22.22 (2) | - | - | - |
| Admitted to burns unit, % (n) | 11.11 (11) | 7.14 (1) | 0 | - | - | - |
| Traditional healer, % (n) | 1.01 (1) | 0 | 22.22 (2) | - | - | - |
| Pharmacy, % (n) | 1.01 (1) | 7.14 (1) | 11.11 (1) | - | - | - |
| Did not seek care, % (n) | 18.18 (18) | 28.57 (4) | 11.11 (1) | - | - | - |
| Not sure, % (n) | 1.01 (1) | 0 | 0 | - | - | - |
| **Number of adults in the household who received the intervention*:** | **N=294** |  |  |  |  |  |
| 1 adult received, % (n) | 71.09 (209) |  |  |  |  |  |
| 2 adults received, % (n) | 22.79 (67) |  |  |  |  |  |
| 3 adults received, % (n) | 4.42 (13) |  |  |  |  |  |
| 4 adults received, % (n) | 1.70 (5) |  |  |  |  |  |
| **How did the household share the burns education:** | | | | | | |
| Did not share, % (n) |  |  | 0.41 (1) |  |  |  |
| Personal messaging, % (n) |  |  | 19.42 (47) |  |  |  |
| Social media, % (n) |  |  | 5.37 (13) |  |  |  |
| In person (word of mouth), % (n) |  |  | 59.09 (143) |  |  |  |
| Meeting (community etc), % (n) |  |  | 4.13 (10) |  |  |  |

**These responses were based on field worker assessments. # Burns data collected at Baseline and Control reflected ever occurrence of burns within the household, while Post-Intervention captured new burns occurring since Baseline. Statistical comparisons were limited to Baseline and Control as the time-limited Post-Intervention data are not directly comparable to cumulative data.*

**Supplementary Table S2. Comparison of Baseline and Control Groups for Caregiver Knowledge and Practice to Exclude Secular Effects**

|  | **Baseline (B)** | **Control (C)** | **p-value** |
| --- | --- | --- | --- |
| **Caregiver practices** | **N=294** | **N=50** |  |
| **Safe zone in place (child prohibited from entering)** | | | |
| None, % (n) | 16.56 (25) | 0 | 0.087 |
| Cooking area, % (n) | 62.25 (92) | 80.00 (12) | 0.172 |
| Bathing area, % (n) | 2.65 (4) | 6.67 (1) | 0.385 |
| All hot water hazard zones (cooking and bathing areas), % (n) | 17.88 (27) | 6.67 (1) | 0.269 |
| Cooking, bathing, and other areas, % (n) | 0.66 (1) | 6.67 (1) | **0.042** |
| Other (Heaters, electrical outlets), % (n) | 0.66 (1) | 6.67 (1) | - |
| This is only during boiling/cooking times, % (n) | 51.66 (78) | 33.33 (5) | 0.176 |
| **Burn risks related to cooking/hot liquid:** | | | |
| Cooking area is inside the house, % (n) | 99.32 (291) | 100 (49) | 0.355 |
| Pot handle inward on stove, % (n) | 57.48 (169) | 48.98 (24) | 0.212 |
| Drinks hot beverages while feeding the child (ever) | 35.88 (94) | 45.00 (18) | 0.266 |
| Caregiver baths a child under 5 years old, % (n) | 84.93 (248) | 81.63 (40) | 0.555 |
| **Child kept >1.5 meters from boiling water or hot meals** | | | |
| Always, % (n) | 68.37 (201) | 62.00 (31) | 0.374 |
| Often (lapses only 1-2 instances per week), % (n) | 7.48 (22) | 20.00 (10) | **0.005** |
| Sometimes (lapses >3 times per week), % (n) | 10.88 (32) | 10.00 (5) | 0.852 |
| Rarely (special occasions), % (n) | 4.08 (12) | 4.00 (2) | 0.978 |
| Never, % (n) | 8.50 (25) | 2.00 (1) | 0.108 |
| **Individual cooks while holding child** | | | |
| Always (routine practice), % (n) | 3.74 (11) | 8.00 (4) | 0.173 |
| Often (once per day), % (n) | 3.74 (11) | 2.00 (1) | 0.535 |
| Sometimes (1-2 instances per week), % (n) | 10.54 (31) | 14.00 (7) | 0.471 |
| Rarely (only special circumstances), % (n) | 5.44 (16) | 6.00 (3) | 0.873 |
| Never, % (n) | 75.85 (223) | 68.00 (34) | 0.238 |
| **Child supervised by an adult** | | | |
| Always, % (n) | 55.10 (162) | 42.00 (21) | 0.086 |
| Often (some parts of at least 5 days of the week), % (n) | 7.82 (23) | 16.00 (8) | 0.062 |
| Sometimes (Once a week or less), % (n) | 10.20 (30) | 22.00 (11) | **0.017** |
| Rarely (Only for special circumstances), % (n) | 4.42 (13) | 2.00 (1) | 0.423 |
| Never, % (n) | 21.77 (64) | 16.00 (8) | 0.354 |
| Caregiver baths a child under 5 years old, % (n) | 84.93 (248) | 81.63 (40) | 0.555 |
| **Burn risks related to bathing for caregivers that bath children:** | **N=248** | **N=40** |  |
| **Method of heating bathwater** | | | |
| Boiled water <5L container (kettle/pot), % (n) | 68.95 (171) | 60.00 (24) | 0.261 |
| Boiled water in >5L container (bucket/metal urn), % (n) | 23.39 (58) | 27.50 (11) | 0.572 |
| Geyser (unregulated), % (n) | 0.81 (2) | 0 | 0.569 |
| Non-regulated geyser combined | 92.34 (229) | 87.50 (35) | 0.304 |
| Other, % (n) | 1.21 (3) | 0 | 0.484 |
| Adds cold water to the bath first, % (n) | 79.84 (198) | 65.00 (26) | **0.036** |
| Checks bathwater temperature before bathing child, % (n) | 99.60 (247) | 100 (40) | 0.687 |
| With Finger, % (n) | 8.50 (21) | 7.50 (3) | 0.832 |
| Hand, % (n) | 70.85 (175) | 62.50 (25) | 0.286 |
| Elbow, % (n) | 17.00 (42) | 30.00 (12) | 0.051 |
| Arm, % (n) | 2.43 (6) | 0 | 0.319 |
| Other (wrist, back of hand), % (n) | 1.21 (3) | 0 | 0.483 |
| **Caregiver knowledge of burn risks** | **N=294** | **N=50** |  |
| **The most common cause of burns is:** | | | |
| Flame, % (n) | 6.80 (20) | 12.00 (6) | 0.199 |
| Hot oil, % (n) | 3.06 (9) | 0 | 0.210 |
| Hot water, % (n) | 83.67 (246) | 80.00 (40) | 0.521 |
| Hot food eg: porridge, % (n) | 2.38 (7) | 4.00 (2) | 0.507 |
| Other (electrical, heaters, stove), % (n) | 2.38 (7) | 2.00 (1) | 0.869 |
| **The most common age at risk of burns:** | | | |
| Less than 1, % (n) | 1.70 (5) | 2.00 (1) | 0.881 |
| 1-5 years, % (n) | 91.84 (270) | 84.00 (42) | 0.078 |
| 6-10 years, % (n) | 5.44 (16) | 12.00 (6) | 0.080 |
| Older than 10 years, % (n) | 0.34 (1) | 0 | 0.680 |
| **The season children most likely to suffer burns:** | | | |
| Summer, % (n) | 5.44 (16) | 2.00 (1) | 0.299 |
| Autumn, % (n) | 3.74 (11) | 4.00 (2) | 0.929 |
| Winter, % (n) | 89.4 (263) | 92.00 (46) | 0.582 |
| Spring, % (n) | 0.68 (2) | 0 | 0.559 |
| **The correct first action to take after a burn:** | | | |
| Apply toothpaste, % (n) | 10.54 (31) | 14.00 (7) | 0.471 |
| Apply something cold/frozen, % (n) | 4.08 (12) | 12.00 (6) | **0.020** |
| Apply oil-based household substance, % (n) | 8.84 (26) | 4.00 (2) | 0.247 |
| Apply food items, % (n) | 8.84 (26) | 2.00 (1) | 0.096 |
| Run cold tap water with ICE for 20 min, % (n) | 7.48 (22) | 4.00 (2) | 0.371 |
| Run cold tap water for 20 min, % (n) | 38.78 (114) | 48.00 (24) | 0.219 |
| After first aid, seek medical care, % (n) | 3.74 (11) | 2.00 (1) | 0.535 |
| Immediately seek medical care without first aid, % (n) | 11.22 (33) | 6.00 (3) | 0.265 |
| Cover the wound with a cloth, % (n) | 3.40 (10) | 4.00 (2) | 0.831 |
| Other (Soap, aloe, traditional medicine, urine, cold air), % (n) | 2.38 (7) | 2.00 (1) | 0.869 |
| Burns cause permanent injuries (True/False), % (n) | 83.56 (244) | 87.76 (43) | 0.457 |
| Burns are preventable (True/False), % (n) | 51.71 (151) | 51.02 (25) | 0.929 |
| Heat-generating appliances should be stored out of child reach (True/False), % (n) | 72.95 (213) | 63.27 (31) | 0.165 |
| Pot handles should be turned inward (True/False), % (n) | 82.53 (241) | 79.59 (39) | 0.619 |

**Supplementary Table S3. Behaviour change post educational intervention at individual level (caregiver matched)**

|  | **Stayed safe** | **Stayed unsafe** | **Became safe** | **Became unsafe** | **p-value*** |
| --- | --- | --- | --- | --- | --- |
| **Burn risks related to bathing for caregivers that bath children:** | | | | | |
| Heating water safely (regulated geyser), % (n) | 2.13 (1) | 93.62 (44) | 2.13 (1) | 2.13 (1) | 0.4795 |
| Adding water to the bath (cold first), % (n) | 82.98 (39) | 0 | 10.64 (5) | 6.38 (3) | 0.7237 |
| Checks water with sensitive body part (wrist, elbow), % (n) | 23.40 (11) | 34.04 (16) | 31.91 (15) | 10.64 (5) | **0.0442** |
| **Safe zone in place (child prohibited from entering)** | | | | | |
| Household designates safe zones, % (n) | 49.09 (27) | 7.27 (4) | 29.09 (16) | 14.55 (8) | 0.1530 |
| Included all hot water hazard zones, % (n) | 0 | 81.48 (22) | 7.41 (2) | 11.11 (3) | 1.0000 |
| Consistently applied (not only risk times), % (n) | 28.57 (10) | 25.71 (9) | 22.86 (8) | 22.86 (8) | 0.8026 |
| Children always/often supervised, % (n) | 52.73 (29) | 10.91 (6) | 23.64 (13) | 12.73 (7) | 0.2636 |
| **Burn risks related to cooking/hot liquid:** | | | | | |
| Consciously turns pot handles inward, % (n) | 52.73 (29) | 7.27 (4) | 38.18 (21) | 1.82 (1) | **0.0014** |
| Doesn't drink hot beverages while feeding child, % (n) | 74.47 (35) | 4.26 (2) | 19.15 (9) | 2.13 (1) | **0.0269** |
| Child kept >1.5m from hot items always, % (n) | 60.00 (33) | 1.82 (1) | 25.45 (14) | 12.73 (7) | 0.1904 |
| Never cooks while carrying child, % (n) | 60.00 (33) | 3.64 (2) | 21.82 (12) | 14.55 (8) | 0.5023 |

**McNemar's Test.*

**Supplementary Table S4. Knowledge change post intervention at individual level (caregiver matched)**

|  | **Consistently correct** | **Consistently incorrect** | **Improved knowledge** | **Declined knowledge** | **p-value*** |
| --- | --- | --- | --- | --- | --- |
| Hot water as the most common type of paediatric burn, % (n) | 74.55 (41) | 3.64 (2) | 20.00 (11) | 1.82 (1) | **0.0094** |
| Children 1-5 years old are most at risk, % (n) | 87.27 (48) | 0 | 7.27 (4) | 5.45 (3) | 1.0000 |
| Winter is the highest risk season, % (n) | 83.64 (46) | 1.82 (1) | 10.91 (6) | 3.64 (2) | 0.2888 |
| Correct First Aid: Cold tap water (20 mins), % (n) | 44.23 (23) | 23.08 (12) | 32.69 (17) | 0 | **0.0001** |
| Burns can lead to permanent injury, % (n) | 83.64 (46) | 0 | 7.27 (4) | 9.09 (5) | 1.0000 |
| Burn injuries are preventable, % (n) | 45.45 (25) | 12.73 (7) | 20.00 (11) | 21.82 (12) | 1.0000 |
| Store heat generating appliances out of child's reach, % (n) | 70.91 (39) | 5.45 (3) | 9.09 (5) | 14.55 (8) | 0.5791 |
| Always turn pot handles inward, % (n) | 78.18 (43) | 0.00 (0) | 18.18 (10) | 3.64 (2) | **0.0433** |

**McNemar's Test.*

**Supplementary Table S5. Behaviour change post educational intervention at household level**

|  | **Stayed safe** | **Stayed unsafe** | **Became safe** | **Became unsafe** | **p-value*** |
| --- | --- | --- | --- | --- | --- |
| **Fieldworker assessments (189 Households)** | | | | | |
| Stove top out of reach (>1.4m), % (n) | 26.46 (50) | 19.05 (36) | 43.39 (82) | 11.11 (21) | **<0.001** |
| Electrical cables for kettle out of reach (>1.4m), % (n) | 49.21 (93) | 10.05 (19) | 22.22 (42) | 18.52 (35) | 0.494 |
| Separate kitchen area, % (n) | 73.54 (139) | 3.17 (6) | 21.16 (40) | 2.12 (4) | **<0.001** |
| **Burn risks related to bathing for caregivers that bath children:** | | | | | |
| Heating water safely (regulated geyser), % (n) | 1.30 (1) | 93.51 (72) | 1.30 (1) | 3.90 (3) | 0.617 |
| Adding water to the bath (cold first), % (n) | 74.03 (57) | 0.00 (0) | 14.29 (11) | 11.69 (9) | 0.823 |
| Checks water with sensitive body part (wrist, elbow), % (n) | 16.88 (13) | 35.06 (27) | 40.26 (31) | 7.79 (6) | **<0.001** |
| **Safe zone in place (child prohibited from entering)** | | | | | |
| Household designates safe zones, % (n) | 35.11 (33) | 13.83 (13) | 32.98 (31) | 18.09 (17) | 0.0606 |
| Included all hot water hazard zones, % (n) | 0.00 (0) | 68.75 (22) | 3.12 (1) | 28.12 (9) | **0.0269** |
| Consistently applied (not only risk times), % (n) | 18.18 (8) | 25.00 (11) | 27.27 (12) | 29.55 (13) | 1.0000 |
| Children always/often supervised, % (n) | 58.51 (55) | 10.64 (10) | 18.09 (17) | 12.77 (12) | 0.4576 |
| **Burn risks related to cooking/hot liquid:** | | | | | |
| Consciously turns pot handles inward, % (n) | 53.19 (50) | 3.19 (3) | 38.30 (36) | 5.32 (5) | **<0.001** |
| Never drinks hot beverages while feeding child, % (n) | 74.12 (63) | 0.00 (0) | 23.53 (20) | 2.35 (2) | **0.0003** |
| Child kept > 1.5m from hot items always, % (n) | 54.26 (51) | 4.26 (4) | 27.66 (26) | 13.83 (13) | 0.0547 |

**McNemar's Test.*

**Supplementary Table S6. Knowledge change post intervention at household level**

|  | **Consistently correct** | **Consistently incorrect** | **Improved knowledge** | **Declined knowledge** | **p-value*** |
| --- | --- | --- | --- | --- | --- |
| Hot water as the most common type of paediatric burn, % (n) | 76.60 (72) | 4.26 (4) | 13.83 (13) | 5.32 (5) | 0.0990 |
| Children 1-5 years old are most at risk, % (n) | 87.23 (82) | 1.06 (1) | 5.32 (5) | 6.38 (6) | 1.0000 |
| Winter is the highest risk season, % (n) | 87.23 (82) | 1.06 (1) | 9.57 (9) | 2.13 (2) | 0.0704 |
| Cold tap water for 20 minutes is the correct first aid, % (n) | 42.86 (39) | 24.18 (22) | 30.77 (28) | 2.20 (2) | **<0.001** |
| Burns can lead to permanent injury, % (n) | 78.72 (74) | 0.00 (0) | 15.96 (15) | 5.32 (5) | **0.0442** |
| Burn injuries are preventable, % (n) | 39.36 (37) | 10.64 (10) | 29.79 (28) | 20.21 (19) | 0.2432 |
| Store heat generating appliances out of child's reach, % (n) | 62.77 (59) | 4.26 (4) | 14.89 (14) | 18.09 (17) | 0.7194 |
| Always turn pot handles inward, % (n) | 77.66 (73) | 0.00 (0) | 20.21 (19) | 2.13 (2) | **0.0005** |

**McNemar's Test.*
